# Diagnostic Roles of Postmortem cTn I and cTn T in Cardiac Death with Special Regard to Myocardial Infarction: A Systematic Literature Review and Meta-Analysis

**DOI:** 10.3390/ijms20133351

**Published:** 2019-07-08

**Authors:** Zhipeng Cao, Mengyang Zhao, Chengyang Xu, Tianyi Zhang, Yuqing Jia, Tianqi Wang, Baoli Zhu

**Affiliations:** 1 Department of Forensic Pathology, School of Forensic Medicine, China Medical University, Shenyang 110122, China; 2 Department of Forensic Genetics and Biology, School of Forensic Medicine, China Medical University, Shenyang 110122, China; 3 The Third Clinical Department of China Medical University, Shenyang 110122, China

**Keywords:** forensic medicine, medical laboratory science, postmortem biochemistry, cardiac death, myocardial infarction, postmortem, cTn I, cTn T, cut-off value, meta-analysis

## Abstract

Background: Cardiac troponin I (cTn I) and cardiac troponin T (cTn T) are currently widely used as diagnostic biomarkers for myocardial injury caused by ischemic heart diseases in clinical and forensic medicine. However, no previous meta-analysis has summarized the diagnostic roles of postmortem cTn I and cTn T. The aim of the present study was to meta-analyze the diagnostic roles of postmortem cTn I and cTn T for cardiac death in forensic medicine, present a systematic review of the previous literature, and determine the postmortem cut-off values of cTn I and cTn T. Methods: We searched multiple databases for the related literature, performed a meta-analysis to investigate the diagnostic roles of postmortem cardiac troponins, and analyzed the receiver operating characteristic (ROC) curve to determine their postmortem cut-off values. Results and Conclusions: The present meta-analysis demonstrated that postmortem cTn I and cTn T levels were increased in pericardial fluid and serum in cardiac death, especially in patients with acute myocardial infarction (AMI). We determined the postmortem cut-off value of cTn I in the pericardial fluid at 86.2 ng/mL, cTn I in serum at 9.5 ng/mL, and cTn T in serum at 8.025 ng/mL.

## 1. Introduction

Although the mortality of cardiovascular death has decreased over the past 20 years, there are still approximately 17 million cardiac deaths in the world every year, nearly 25% of which are sudden cardiac death (SCD) [1]. Death caused by cardiovascular diseases, especially myocardial infarction (MI), is a global public health problem, which accounts for large proportions of the routine work of forensic practice [2,3,4,5]. However, typical morphological findings are sometimes quite limited in certain cardiac deaths during autopsies and histological investigations [5,6]. Thus, objective evidence for the diagnosis of cardiac death is both imperative and urgent. Postmortem biochemical analysis of several biomarkers or indicators, such as cardiac troponins, creatine kinase MB (CK-MB), and N-terminal proBNP (NT-proBNP), plays a significant role in the postmortem diagnosis of cardiac death and may provide an auxiliary diagnosis basis for SCD [7,8].

**Figure 1:**
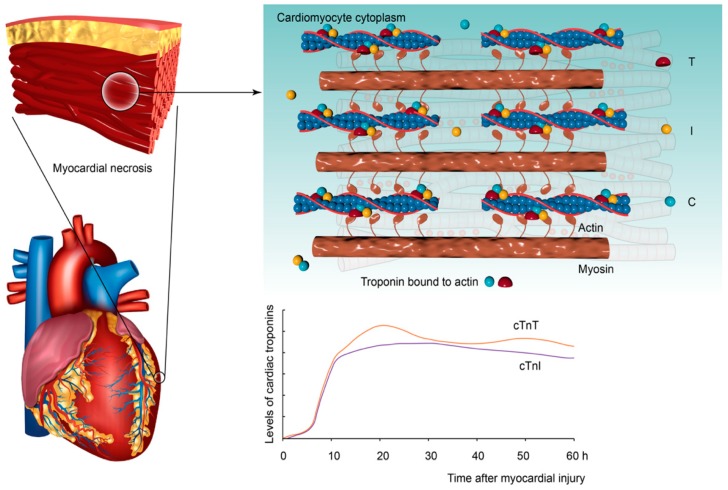
Schematic diagram of cardiac troponin composition.

Cardiac troponin is a regulatory protein of striated muscle contraction that is present as a complex in the thin filaments of myofibrils and has three subunits: cardiac troponin T (cTn T), cardiac troponin I (cTn I), and cardiac troponin C (cTn C) [9]. cTn I and cTn T are cardiac-specific and highly sensitive biomarkers that reflect myocardial damage [10,11]. They can be detected in the blood 3–4 h after myocardial infarction, peak after 16–18 h, and last for up to two weeks in the blood [9,12,13,14] (Figure 1). Due to the high sensitivity and specificity of myocardial injury, cardiac troponins are recommended by the European Society of Cardiology and the American College of Cardiology to be the primary choice and significant reference for the diagnosis of myocardial infarction [15,16,17]. The levels of cardiac troponins not only have the effect of diagnosing myocardial infarction but also can be used to assess the severity of infarction and prognosis in clinical medicine [18,19,20,21].

Recently, postmortem biochemical analyses of cTn I and cTn T in different bodily fluids were conducted by several forensic teams, and cardiac troponins have proved to be of great value in the postmortem diagnosis of cardiac death [22]. However, the accuracy and specificity of postmortem levels of cardiac troponins in different sampling sites are reported to be inconsistent [22,23]. A majority of the related studies demonstrated that postmortem biochemical analysis of cardiac troponins is useful in the diagnosis of cardiac death, but its specificity and diagnostic value are suspect [24]. No previous meta-analysis has objectively summarized the diagnostic roles of postmortem cTn I and cTn T for cardiac death, and the postmortem cut-off values of cTn I and cTn T are imperative as well. Therefore, the present study systematically reviewed and meta-analyzed the screened literature about postmortem biochemical analysis of cTn I and cTn T to investigate their diagnostic roles in cardiac death, especially in myocardial infarction. The postmortem cut-off values of cTn I and cTn T were also determined using receiver operating characteristic (ROC) curve analysis.

## 2. Results

### 2.1. Included Literature

A total of 301 studies were collected using the search strategy, of which there were 185 works found in PubMed, 50 in Embase, 8 in the Cochrane Library, 5 in the China National Knowledge Infrastructure (CNKI), 10 in the China Biomedical Literature Database (CBM), and 43 in Wanfang Data. Of the 301 articles, 35 records were excluded due to duplication, and finally, 13 studies were included in the present meta-analysis after screening the full text carefully according to the inclusion and exclusion criteria.

### 2.2. Characteristics of Involved Studies

The characteristics of these 13 included studies are presented in Table 1, including the author, the published year, the age range, the postmortem interval (PMI), analyzed samples, and the analyzed biomarkers. In our original design, we intended to compare the differences of cTn I and cTn T in pericardial fluid and serum, respectively, between the cardiac death and control groups. Due to the insufficient study, finally, we analyzed six groups of comparative data in meta-analysis practice, which are as described below.

Some of the included studies investigated both cTn I and cTn T levels in different postmortem samples. Four of the included 13 studies (Pérez-Cárceles et al., 2004 [27]; Martínez Díaz et al., 2005 [28]; Batalis et al., 2010 [30]; and Sapouna et al., 2013 [33]) focused on the discrepancies of postmortem cTn I concentrations in pericardial fluid between the cardiac death group and the group which involved other causes of death, and it was found that the cardiac death in all four studies was due to myocardial infarction. Two of the included 13 studies (Remmer et al., 2013 [32] and González-Herrera et al., 2016 [34]) focused on the comparisons of postmortem cTn T concentrations in pericardial fluid between the cardiac death and control groups, and only one of these two studies (González-Herrera et al., 2016 [34]) explicitly stated that the cause of death included myocardial infarction. Therefore, data analysis regarding cTn T in pericardial fluid was not performed between the myocardial infarction and control groups. For the postmortem cTn I levels in serum, five of the included 13 studies (Pérez-Cárceles et al., 2004 [27]; Martínez Díaz et al., 2005 [28]; Batalis et al., 2010 [30]; Sun et al., 2011 [31]; and Carvajal-Zarrabal et al., 2017 [35]) compared the differences between the cardiac death and control groups, of which, four studies (Pérez-Cárceles et al., 2004 [27]; Martínez Díaz et al., 2005 [28]; Batalis et al., 2010 [30]; and Sun et al., 2011 [31]) recruited patients who died of myocardial infarction. For the postmortem cTn T levels in serum, senen of the included 13 studies (Zhu et al., 2003 [25]; Ellingsen et al., 2004 [26]; Khalifa et al., 2006 [29]; Remmer et al., 2013 [32]; Carvajal-Zarrabal et al., 2017 [35]; Beausire et al., 2018 [36]; and Rahimi et al., 2018 [24]) focused on the comparisons of postmortem cTn T concentrations in serum between the cardiac death and noncardiac death groups, and four of the seven studies (Zhu et al., 2003 [25]; Ellingsen et al., 2004 [26]; Khalifa et al., 2006 [29]; and Rahimi et al., 2018 [24]) used cases in which the patients died of myocardial infarction (Table 2).

### 2.3. Meta-Analysis

**Figure 2:**
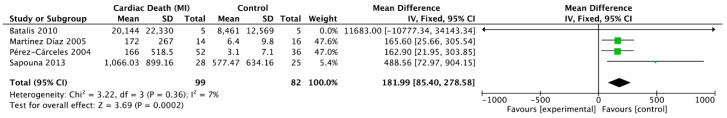
Forest plot of cTn I in pericardial fluid between cardiac death and control groups.

**Figure 3:**
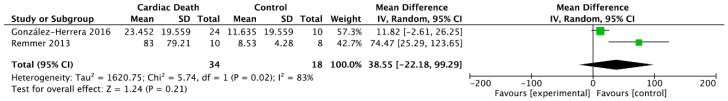
Forest plot of cTn T in pericardial fluid between cardiac death and control groups.

The meta-analysis was performed based on the above description. Heterogeneity was significant when *p* < 0.05 and/or I^2^ > 50%, and a random-effect model was used; if not, a fixed-effect model was used. In the six groups mentioned above, the fixed-effect model was applicable for studies on the postmortem cTn I concentrations in the pericardial fluid between the cardiac death (myocardial infarction) and control groups, and the random-effect model was used to analyze data in the other groups. Compared with the control group, the level of cTn I in the pericardial fluid was higher than in the cardiac death group (weighted mean difference (WMD) = 181.99, 95% confidence interval (CI) = 85.40–278.58, *p* = 0.0002; I^2^ = 7%, *p* = 0.36) (Figure 2). However, no significant difference was found for the postmortem cTn T in the pericardial fluid between the cardiac death and control groups (WMD = 38.55, 95% CI = −22.18 to 99.29, *p* = 0.21; I^2^ = 83%, *p* = 0.02) (Figure 3).

**Figure 4:**
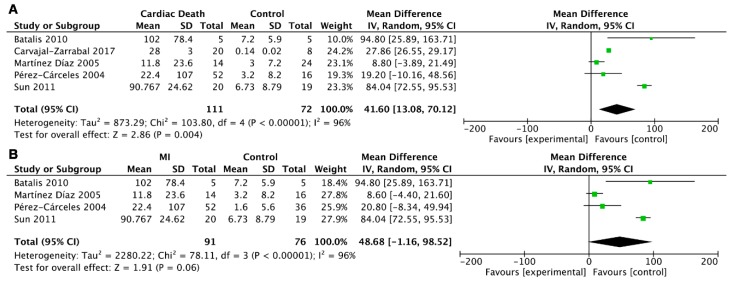
Forest plot of cTn I in serum: (**A**) comparison of serum cTn I between cardiac death and control groups; (**B**) comparison of serum cTn I between the myocardial infarction (MI) and control groups.

**Figure 5:**
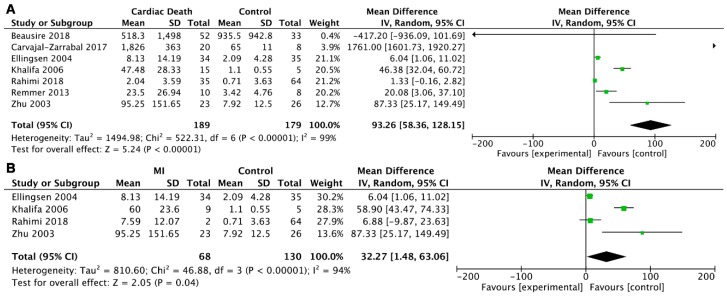
Forest plot of cTn T in serum: (**A**) comparison of serum cTn T between the cardiac death and control groups; (**B**) comparison of serum cTn T between the MI and control groups.

To investigate the diagnostic roles of postmortem cTn I and cTn T concentrations in serum, we carried out a meta-analysis of another four groups. Statistically significant differences were found when comparing serum cTn I and cTn T between the cardiac death and control groups (WMD = 41.60, 95% CI = 13.08–70.12, *p* = 0.004; I^2^ = 96%, *p* < 0.00001; WMD = 93.26, 95% CI = 58.36–128.15, *p* < 0.00001; I^2^ = 99%, *p* < 0.00001). Furthermore, we analyzed the serum cTn I and cTn T levels in myocardial infarction, and no significant difference was found for serum cTn I (WMD = 48.68, 95% CI = −1.16 to 98.52, *p* = 0.06; I^2^ = 96%, *p* < 0.00001), but there was a statistically significant difference for serum cTn T (WMD = 32.27, 95% CI = 1.48–63.06, *p* = 0.04; I^2^ = 94%, *p* < 0.00001) (Figure 4; Figure 5).

### 2.4. Speculation of Postmortem Cut-off Values of cTn I and cTn T by ROC

**Figure 6:**
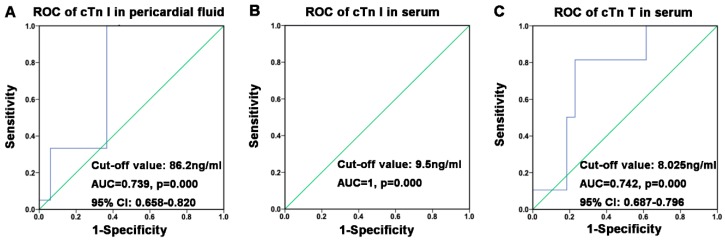
Receiver operating characteristic (ROC) curve of cardiac troponins in pericardial fluid and serum: (**A**) ROC of cTn I in pericardial fluid; (**B**) ROC of cTn I in serum; (**C**) ROC of cTn T in serum.

The specificity and sensitivity of cardiac troponins for diagnosing cardiac death were calculated based on the mean concentrations of cTn I in pericardial fluid, cTn I in serum, and cTn T in serum between the cardiac death and control groups. The speculated cut-off values, the area under the curve (AUC), *p*-value, and 95% CI are shown Figure 6.

## 3. Discussion

Postmortem diagnosis of cardiac death mainly depends on autopsy and histological examination, but typical morphological findings are sometimes quite limited [5,6]. Due to their high cardiac sensitivity and specificity, small molecular weight, difficulty in decomposition, long half-life, and reflection of myocardial damage, cTn I and cTn T have been objectively considered to be the routine auxiliary biomarkers for postmortem diagnosis of cardiac death [7,8,37]. The present study not only meta-analyzed the diagnostic roles of postmortem cTn I and cTn T in cardiac death but also reviewed the following issues, which should be noted by forensic pathologists.

### 3.1. The Concentration Changes of Cardiac Troponin in Antemortem and Postmortem Samples

Because of autolysis, microbial degradation, normal metabolic arrest, and excretion, cTn I and cTn T levels in postmortem bodily fluids are quite different from those in antemortem samples [22,38]. Postmortem cTn I and cTn T levels in serum from different sampling sites were demonstrated to be higher than the antemortem ones, independent of the cause of death [23,26,30,34,38]. Postmortem cardiac troponin levels in the pericardial fluid are also higher than they are in antemortem pericardial fluid [34]. Therefore, the assessment of postmortem cardiac troponin levels cannot be equated with antemortem ones nor with the diagnostic cut-off value in clinical medicine.

Moreover, samples from most corpses may have different degrees of hemolysis in forensic practice [30]. Frozen postmortem samples used for postmortem biochemical analysis should be avoided because freezing may cause severe hemolysis and affect the concentration changes of biochemical indicators in the bodily fluid. We suggest that the corpses should be refrigerated because refrigeration can delay the autolysis of bodies and at the same time reduce the degree of hemolysis [22,39].

### 3.2. Stability of Postmortem cTn I and cTn T with Regard to Cardiopulmonary Resuscitation, Age, Gender, and PMI

Cardiopulmonary resuscitation is a strong external chest compression that patients receive at the end-stage of life. Similar to chest trauma, cardiopulmonary resuscitation may cause damage to the myocardium. However, related studies have shown that cardiopulmonary resuscitation has no effect on cardiac troponin levels [22,23,34,40,41]. In addition, postmortem cardiac troponin levels are demonstrated to not be associated with age or gender [22,32,33,34,42]. Therefore, there is no need for forensic pathologists to consider the impact of cardiopulmonary resuscitation, age, and gender when analyzing cardiac troponin levels.

Cardiac troponin concentrations in both serum and pericardial fluid had a postmortem time-dependent increase, especially when PMI was greater than 48 h [32]. However, no statistical differences were observed in cTn I and cTn T levels between samples with a PMI of 4–24 h and a PMI of 25–48 h, which indicated that both cTn I and cTn T had postmortem stability within 48 h after death [23,26]. Samples with a long PMI have limited application in postmortem biochemical analysis due to few studies having focused on postmortem redistribution and the impact of postmortem changes such as hemolysis. As a result, further research is needed on the biochemical analysis of postmortem samples with a long PMI.

### 3.3. Cardiac Troponins in Different Sampling Sites

Postmortem cTn I and cTn T levels were closely correlated with different sampling sites and the cause of death [32,43]. Among different peripheral blood samples, iliac vein or femoral vein blood, which is located far from the heart, was demonstrated to be more suitable for postmortem biochemical analysis [23,30,44,45]. Furthermore, postmortem cTn I and cTn T concentrations in the pericardial fluid have been demonstrated to be higher than those in postmortem peripheral venous blood. Studies have even found that cTn I and cTn T concentrations in pericardial fluid are up to 100 times higher than those in serum obtained from femoral venous blood [23,27,28,30,32,44,45]. Pericardial fluid is the closest to myocardium and this may explain why pericardial fluid has higher postmortem levels of cTn I and cTn T. Chemicals and other markers may be secreted directly into pericardial fluid after myocardial injury. Therefore, the concentration of these chemicals increases first in pericardial fluid and then in peripheral blood [30,33,40,41,43,46]. Pericardial fluid is present in a closed serosa cavity and is not vulnerable to contamination and postmortem changes such as autolysis and spoilage [47,48]. In addition, pericardial fluid is easy to obtain during forensic autopsy and is currently used as a substitute for serum in postmortem biochemical analysis [49,50,51]. It is reported that pericardial fluid is the best sampling site for cardiac troponin detection of myocardial injury, with a sensitivity of 89.1% and a specificity of 95.2% [43]. Therefore, it seems that pericardial fluid is better than serum for postmortem biochemical analysis, especially for hemolyzed samples.

In the present study, we performed a meta-analysis on cTn I and cTn T levels in both pericardial fluid and serum among cardiac death and control groups, respectively. Some of the included studies analyzed cardiac troponin concentrations in serum from different anatomic sites. Based on the results of previous studies, the present meta-analysis suggests preferential use of peripheral blood such as iliac or femoral vein blood, which is further away from the heart.

### 3.4. Cardiac Troponins in Cardiac Death

The results of our meta-analysis objectively revealed that the cTn I level in both pericardial fluid and serum and cTn T levels in serum were statistically higher in the cardiac death group than those in the control group. However, there were only two studies with different analysis methods of the postmortem level of cTn T in pericardial fluid. Although no statistical differences were found in the meta-analysis of the only two studies which focused on the postmortem level of cTn T in pericardial fluid, both of the studies showed statistically elevated pericardial cTn T in the cardiac death group. Similar to clinical studies, individuals who died of myocardial infarction showed the highest concentration of cTn I and cTn T among different causes of death [4,27,28,29,40]. The present meta-analysis showed that cTn I concentration in pericardial fluid and cTn T concentration in serum were statistically higher in individuals with myocardial infarction. Therefore, postmortem biochemical analysis of cardiac troponins is of great significance for the diagnosis of cardiac death, especially acute myocardial infarction (AMI).

It is worth noting that elevated cardiac troponin levels can also be observed in other causes of death in forensic practice, such as hyperthermia, methamphetamine abuse, carbon monoxide poisoning, electric shock, psychotropic drug poisoning, pulmonary embolism, end-stage renal failure, and cerebrovascular disease [22,26,27,32,42,45,52]. In addition, normal troponin levels cannot rule out the possibility of cardiac death because cardiac troponins cannot be detected in peripheral blood until about 3 h after myocardial injury [26]. Therefore, postmortem biochemical analysis of cardiac troponins is an auxiliary diagnosis basis, but the diagnosis of cardiac death should not only be based on the levels of postmortem cardiac troponins.

### 3.5. Cut-off Values of Postmortem cTn I and cTn T

Some of the previous studies reported the postmortem cut-off values of cardiac troponin in different bodily fluids, but they had different values based on different sampling sites and PMI. For the cut-off value in different sampling sites, Aissaoui et al. analyzed the concentration of cTn I in the cardiac blood, peripheral blood, and pericardial fluid in individuals with or without myocardial injury by ROC and reported the cut-off value at 70.66 ng/mL in cardiac blood, 11 ng/mL in peripheral blood, and 108 ng/mL in pericardial fluid [43]. González-Herrera et al. reported that the postmortem cut-off value of cTn T was 250 ng/mL in serum (femoral blood) and 3200 ng/mL in pericardial fluid [34]. The cut-off value of cTn I in pericardial fluid was determined to be 1.418 ng/mL [27]. For cases with different PMIs, Zhu et al. reported the cut-off value of cTn T in peripheral blood (iliac blood) at 0.2 ng/mL (PMI < 12 h) and 0.6 ng/mL (PMI: 12–48 h), and the cut-off value of cTn T in pericardial fluid at 10 ng/mL (PMI < 12 h) and 100 ng/mL (PMI: 12–48 h) [44,45]. Currently, there is no established postmortem cut-off value for cardiac troponins based on the data of a large sample size. The ROC analysis of the present study revealed a postmortem cut-off value of cTn I in pericardial fluid at 86.2 ng/mL, cTn I in serum at 9.5 ng/mL, and cTn T in serum at 8.025 ng/mL, results which were similar to those of a previous study [43]. The distinct postmortem cardiac troponin cut-off values may vary predominantly due to different PMI and analysis methods. Because there were only two studies involving cTn T in pericardial fluid, we did not perform ROC analysis for cTn T in pericardial fluid.

### 3.6. Analysis Methods of Cardiac Troponins

The detection method for cardiac troponins has been developed with the improvement of new laboratory technologies. Recently, many detection methods have been developed for quantitative analysis of cardiac troponins, including an enzyme-linked immunosorbent assay (ELISA), chemiluminescent immunoassay, fluoroimmunoassays, electrical detections, surface-plasmon-resonance-based detection, colorimetric protein array, and point-of-care testing (POCT) [13,53,54,55,56,57,58,59,60]. Each detection method has its own advantages and disadvantages, causing potential problems for postmortem biochemical analysis. Currently, there is no golden testing method or standard reference value for postmortem biochemical analysis of cardiac troponins in forensic science. Therefore, there is no comparability for the results. To solve this problem, we suggest that each postmortem biochemical testing laboratory in forensic medicine should establish a database and reference values based on their own biochemical analysis methods. During the testing process, attention should be paid to the trends in differences across different causes of death, rather than the absolute concentration value.

As mentioned above, there are currently many immunological assays for the detection of cardiac troponin, but these methods require expensive reagents and equipment [61]. POCT detection based on ELISA, chemiluminescence, and other techniques can be used for a rapid cardiac troponin assay, reducing the testing time and cost of diagnostic evaluation [4,41,62].

## 4. Materials and Methods

### 4.1. Strategy of Literature Search

**Figure 7:**
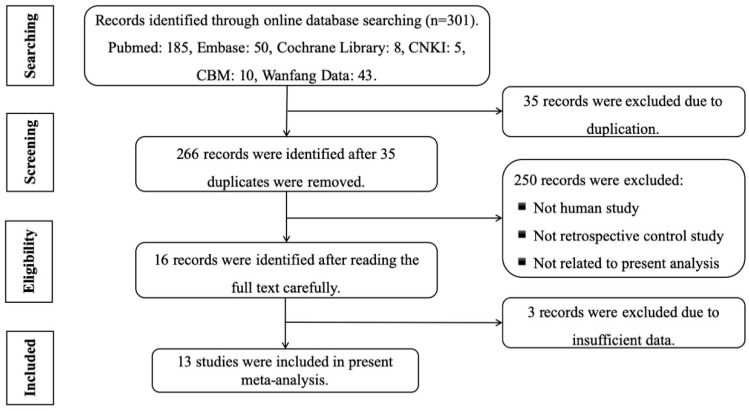
Flow diagram of literature search process.

The online databases Pubmed, Embase, Cochrane Library, CNKI, CBM, and Wanfang Data were comprehensively searched by two investigators (Cao and Zhao) independently to collect the available retrospective control studies up to the year 2000, and the latest search time was May 1, 2019. A similar search strategy was used as that detailed by Barberi et al. [22]. Take Pubmed as an example: keywords related to the study objective included in the search string were cardiac troponins, forensic postmortem, and subsequently, the following refined PubMed/MeSH search words: ((“heart”[MeSH Terms] OR “cardiac”[All Fields]) AND (“troponin”[MeSH Terms] OR “troponins”[All Fields]) AND “forensic”[All Fields] AND “postmortem”[All Fields]). Then, the unrelated or improper studies were excluded by scanning the abstracts, and the data were extracted from included studies by reading the full texts carefully. Additionally, the other literature mentioned in the reviews or references was also searched and browsed. Based on the original design of this meta-analysis, we intended to compare the discrepancies in concentrations of cTn I and cTn T in pericardial fluid and serum in individuals suffering from cardiac death or myocardial infarction (in some studies, the former equals the latter) with the control studies, respectively (Figure 7).

### 4.2. Inclusion Criteria

Studies with all of the following criteria were included in the systematic review and meta-analysis: (1) retrospective control study and categorical sample contents; (2) study of the postmortem cTn I or cTn T value in cardiac death; and (3) enough data were provided to obtain the WMD and the corresponding 95% CI or *p*-values for diagnosis outcomes.

### 4.3. Exclusion Criteria

Studies with any of the following criteria were excluded: (1) repeated published studies; (2) noncontrolled studies; (3) poor-quality or statistically illogical studies; (4) not involved in the role of postmortem cTn I or cTn T in cardiac death; and (5) provided data were insufficient to obtain the WMD or 95% CIs.

### 4.4. Data Abstraction and Quality Assessment

Two investigators extracted the following data after reading original studies carefully and independently: name of the first author, publication year, sample characteristics (size, age, and PMI), and mean and standard deviation (SD). Furthermore, in the case of some studies in which the mean or SD were not provided directly, two investigators counted out the corresponding results independently by reliable statistical methods. A third investigator was needed if there was discrepancy after cross-checking.

The quality assessment of the included studies was conducted with the Newcastle–Ottawa Quality Assessment Scale (NOS), and the studies with NOS scores ≥ 7 were considered to be high-quality studies [63]. The evaluation contents include: (1) Selection of the included studies: ① case definition adequate (the cause of death was diagnosed by at least two independent pathologists according to autopsy findings, histological investigations, and other lab investigations), ② representativeness of the cases, ③ selection of controls, ④ definition of controls (1 point for each item, 4 points in total). (2) Comparability of cases and controls on the basis of the design or analysis: ⑤ study controls for cardiac death and noncardiac death cases, ⑥ study controls for PMI < 48 h (1 point for each item, 2 points in total). (3) Exposure: ⑦ ascertainment of exposure, ⑧ same method of ascertainment for cases and controls, ⑨ nonresponse rate (1 point for each item, 3 points in total) (Table 3).

### 4.5. Statistical Analysis

A meta-analysis was performed using Review Manager version 5.3 software, and the diagnosis results were evaluated using WMD and 95% CI of included studies. Heterogeneity was assessed across all studies by Cochran’s Q test and Higgins’s I^2^. The heterogeneity was significant if *p* < 0.05 and/or I^2^ > 50%, and in this case, the random-effect model was used. Otherwise, the fixed-effect model was used. However, the funnel plot and sensitivity analysis were not carried out due to the low number of included studies. All *p*-values were two sided, and *p* < 0.05 was considered to be statistically significant.

The mean values of postmortem cTn I and cTn T in pericardial fluid and serum were used to plot an ROC by SPSS 22.0 for Mac (SPSS Inc., Chicago, IL, USA) for cut-off values.

## 5. Conclusions and Perspective

The present meta-analysis demonstrated that postmortem cTn I and cTn T levels are increased in both pericardial fluid and serum in cardiac death, especially in individuals with myocardial infarction. We determined the postmortem cut-off value of cTn I in the pericardial fluid at 86.2 ng/mL, cTn I in serum at 9.5 ng/mL, and cTn T in serum at 8.025 ng/mL. For more than 20 years, research has outlined the significance of postmortem analysis of cardiac troponins in cardiac death, particularly in myocardial infarction. It has been proved that biochemical analysis of cardiac troponins can be used not only as an indicator to reflect myocardial injury in clinical medicine but also as an auxiliary diagnosis basis for SCD in forensic medicine, especially for those without morphological findings.

However, forensic pathologists should be aware of the following issues. First, corpses in cryopreservation can have severe hemolysis, and postmortem biochemical analysis will be affected. Therefore, cryopreservation should be avoided for corpses and postmortem samples used for postmortem biochemical analysis. Second, the sample collection time should be within 48 h after death to avoid the impact of PMI on postmortem cardiac troponin analysis. Third, we recommend that the pericardial fluid and femoral veins or external iliac veins are the best sampling sites for biochemical analysis. Fourth, normal cardiac troponin levels cannot rule out the possibility of cardiac death, and vice versa, and elevated cardiac troponin levels cannot determine cardiac death but can help to analyze the cause of death. Finally, each forensic laboratory should establish its own reference value of cardiac troponin levels.

## Figures and Tables

**Table 1:** The characteristics of the included studies.

Author	Year	Age	PMI (h)	Analyzed Sample(s)	Analyzed Biomarker(s)
Zhu et al. [25]	2003	0–94	<48	Serum	Cardiac troponin T (cTn T)
Ellingsen et al. [26]	2004	4–92	3–75	Serum	cTn T
Pérez-Cárceles et al. [27]	2004	14–87	1–29	Pericardial fluid and serum	cardiac troponin I (cTn I)
Martínez Díaz et al. [28]	2005	12–87	2–16	Pericardial fluid and serum	cTn I
Khalifa et al. [29]	2006	—	6–20	Serum	cTn T
Batalis et al. [30]	2010	—	<24	Pericardial fluid and serum	cTn I
Sun et al. [31]	2011	0.5–76	<288	Serum	cTn I
Remmer et al. [32]	2013	25–54	8–141	Pericardial fluid and serum	cTn T
Sapouna et al. [33]	2013	13–94	8–48	Pericardial fluid	cTn I
González-Herrera et al. [34]	2016	27–95	5–34	Pericardial fluid and serum	cTn T (high-sensitivity assay)
Carvajal-Zarrabal et al. [35]	2017	24–74	<8	Serum	cTn I and cTn T
Beausire et al. [36]	2018	15–75	<72	Serum	cTn T (high-sensitivity assay)
Rahimi et al. [24]	2018	18–50	<24	Serum	cTnT

**Table 2:** The main results of subgroup analysis.

Biomarker	Cause of Death	Included Studies	Weighted Mean Difference (WMD) (95% CI)	*p*	I^2^	*p*-Value of Heterogeneity
cTn I in pericardial fluid	Cardiac death (myocardial infarction)	4	181.99 (85.40, 278.58)	0.0002	7%	0.36
cTn T in pericardial fluid	Cardiac death	2	38.55 (−22.18, 99.29)	0.21	83%	0.02
cTn I in serum	Cardiac death	5	41.60 (13.08, 70.12)	0.004	96%	<0.00001
	Myocardial infarction	4	48.68 (−1.16, 98.52)	0.06	96%	<0.00001
cTn T in serum	Cardiac death	7	93.26 (58.36, 128.15)	<0.00001	99%	<0.00001
	Myocardial infarction	4	32.27 (1.48, 63.06)	0.04	94%	<0.00001

**Table 3:** Quality assessment of included studies.

Study	Quality indicators from the Newcastle–Ottawa Scale									
	Selection				Comparable		Outcome Assessment			Scores
	①	②	③	④	⑤	⑥	⑦	⑧	⑨	
Zhu et al., 2003 [25]	1	1	1	1	1	1	1	1		8
Ellingsen et al., 2004 [26]	1	1	1	1	1		1	1		7
Pérez-Cárceles et al., 2004 [27]	1	1	1	1	1	1	1	1		8
Martínez Díaz et al., 2005 [28]	1	1	1	1	1	1	1	1		8
Khalifa et al., 2006 [29]	1	1	1	1	1	1	1	1		8
Batalis et al., 2010 [30]	1	1	1	1	1	1	1	1		8
Sun et al., 2011 [31]	1	1	1	1	1		1	1		7
Remmer et al., 2013 [32]	1	1	1	1	1		1	1		7
Sapouna et al., 2013 [33]	1	1	1	1	1	1	1	1		8
González-Herrera et al., 2016 [34]	1	1	1	1	1	1	1	1		8
Carvajal-Zarrabal et al., 2017 [35]	1	1	1	1	1	1	1	1		8
Beausire et al., 2018 [36]	1	1	1	1	1		1	1		7
Rahimi et al., 2018 [24]	1	1	1	1	1	1	1	1		8

## Abbreviations

ACS	Acute coronary syndrome
AMI	Acute myocardial infarction
AUC	Area under the curve
NT-proBNP	N-terminal proBNP
CBM	China Biomedical Literature Database
CI	Confidence interval
CK-MB	Creatine kinase MB
CNKI	China National Knowledge Infrastructure
cTn I	Cardiac troponin I
cTn T	Cardiac troponin T
ELISA	Enzyme-linked immunosorbent assay
MI	Myocardial infarction
PMI	Postmortem interval
POCT	Point-of-care testing
ROC	Receiver operating characteristic curve
SCD	Sudden cardiac death
WMD	Weighted mean difference
